# Nanotechnology-Enabled Precision Therapy for Lung Cancer in Never-Smokers

**DOI:** 10.3390/pharmaceutics18020161

**Published:** 2026-01-26

**Authors:** Cristian Cojocaru, Adina Magdalena Țurcanu, Ruxandra Cojocaru, Elena Cojocaru

**Affiliations:** Grigore T. Popa University of Medicine and Pharmacy, 700115 Iasi, Romania; cristian.cojocaru@umfiasi.ro (C.C.); mg-rom-34267@students.umfiasi.ro (R.C.); elena.cojocaruu@umfiasi.ro (E.C.)

**Keywords:** lung cancer, never-smokers, nanocarriers, targeted therapy, CNS delivery

## Abstract

Lung cancer in never-smokers (LCINS) represents a distinct clinical entity driven by dominant oncogenic alterations and characterized by a low tumor mutational burden. Although tyrosine kinase inhibitors (TKIs) achieve high initial response rates, their long-term efficacy is limited by suboptimal pharmacokinetics, restricted central nervous system (CNS) penetration, tumor microenvironment barriers, and acquired resistance. In this review, we critically assess the current state of nanotechnology-assisted drug delivery systems for LCINS, with a primary focus on how rationally designed nanocarriers can overcome biological barriers, enable molecular subtype-specific therapeutic strategies, and address mechanisms that limit clinical efficacy and durability of response. We conducted a structured literature search using PubMed and Web of Science (January 2022 to November 2025), focusing on primary studies reporting the preparation, physicochemical properties, and therapeutic performance of nanocarriers in in vitro and in vivo models, as well as available pharmacokinetic and clinical data. LCINS is characterized by inefficient vasculature, high extracellular matrix density, active efflux transporters, and immunosuppressive niches, and is frequently complicated by brain metastases. Nanocarrier-based platforms can enhance aqueous solubility, prolong systemic circulation, and improve tumor or CNS targeting. Co-delivery systems combining TKIs with nucleic acid-based therapeutics, together with stimuli-responsive platforms, offer the potential for simultaneous modulation of multiple oncogenic pathways and partial mitigation of resistance mechanisms. In summary, nanotechnology provides a promising strategy to improve both the efficacy and specificity of targeted therapies in LCINS. Successful clinical translation will depend on biologically aligned carrier–payload combinations, scalable and reproducible manufacturing processes, and biomarker-guided patient selection.

## 1. Introduction

Lung cancer in never-smokers (LCINS) represents a distinct biological entity within non-small-cell lung cancer (NSCLC) [1,2]. In contrast to smoking-associated NSCLC, which accumulates numerous low-frequency mutations following prolonged exposure to tobacco carcinogens, LCINS is typically driven by a limited number of dominant oncogenic driver events. The most frequent alterations include activating epidermal growth factor receptor (EGFR) mutations (exon 19 deletions and L858R substitutions), fusions involving anaplastic lymphoma kinase (ALK), c-ros oncogene 1 receptor tyrosine kinase (ROS1), rearranged during transfection (RET), mesenchymal–epithelial transition (MET) exon 14 skipping, and human epidermal growth factor receptor (HER) 2 exon 20 alterations [3,4,5]. The high allele frequency of these drivers underlies the concept of “oncogene addiction,” rendering LCINS highly sensitive to tyrosine kinase inhibitors (TKIs). Nevertheless, durable clinical responses remain uncommon, largely because pharmacokinetic constraints and tumor microenvironmental barriers limit effective drug exposure. Many TKIs are hydrophobic, exhibit poor aqueous solubility, undergo rapid hepatic metabolism, and fail to achieve sufficient penetration into solid tumors or the central nervous system (CNS) [6].

The blood–brain barrier (BBB) severely restricts drug entry into the CNS, a frequent and clinically significant site of metastasis in LCINS. CNS involvement is associated with substantial morbidity and reduced survival, underscoring the therapeutic challenge posed by intracranial disease. Because the BBB limits the penetration of most targeted therapies, nanocarrier-based strategies designed to enhance brain delivery constitute a central focus of this review. Sublethal drug exposure within residual intracranial lesions promotes the emergence of resistance through secondary mutations, activation of bypass signaling pathways, and lineage plasticity. Consequently, the principal clinical limitation in LCINS is often not target specificity but insufficient drug delivery to sanctuary sites. Accordingly, CNS-directed delivery strategies are positioned as a core theme of this review rather than a peripheral consideration, consistent with recent evidence highlighting the high incidence of brain metastases and the critical role of the BBB in LCINS [7].

Nanotechnology offers versatile tools to modulate the pharmacokinetics and biodistribution of anticancer agents. Nanocarriers are engineered structures typically ranging from 1 to 200 nm in size that can encapsulate hydrophobic drugs, protect them from degradation, and prolong systemic circulation, thereby facilitating targeted delivery to tumors and metastatic sites. Key physicochemical parameters—including size, shape, surface charge, and ligand density—can be precisely tuned to enable receptor-mediated transcytosis, controlled drug release, and improved tissue penetration [8].

Co-encapsulation of multiple therapeutic agents—such as small interfering ribonucleic acid (siRNA) or clustered regularly interspaced short palindromic repeats (CRISPR)/Cas9 systems—enables the simultaneous targeting of primary oncogenic drivers and associated bypass signaling pathways. In addition, stimuli-responsive nanomaterials can be engineered to trigger controlled drug release in response to specific features of the tumor microenvironment, including pH, redox status, or enzymatic activity [9].

This review integrates recent advances in cancer biology with innovations in nanomedicine. It focuses on microenvironmental barriers to drug delivery, comparative analysis of nanocarrier platforms, molecular subtype—specific targeting strategies, stimuli-responsive systems, and approaches to mitigate therapeutic resistance. Safety, immunogenicity, and translational considerations are also addressed, with particular emphasis on quantitative performance metrics, recent primary studies, and practical insights relevant to clinical translation.

## 2. Biological and Microenvironmental Foundations

### 2.1. Oncogenic Drivers and Addiction

LCINS tumors are dominated by high-impact genomic alterations. Activating EGFR exon 19 deletions and L858R mutations occur in approximately 15–55% of cases depending on ethnicity; ALK fusions are present in 3–7%; ROS1 and RET fusions each account for approximately 1–2%; MET exon 14 skipping mutations are observed in 3–4%; and HER2 exon 20 insertions occur in about 2% of never-smokers [6].

### 2.2. Microenvironmental Delivery Barriers

Drug distribution within tumors is governed by a combination of physical and biological barriers. In LCINS, the extracellular matrix is dense with collagen-rich components and is associated with elevated interstitial fluid pressure, which limits effective convective transport. Tumor vasculature is also heterogeneous: in some LCINS lesions, the endothelial lining is discontinuous and highly permeable, whereas in others tight junctions remain largely intact, rendering the enhanced permeability and retention (EPR) effect weak or inconsistent [10].

Cells forming the BBB express active efflux transporters, including P-glycoprotein (P-gp) and breast cancer resistance protein, which pump therapeutic compounds back into the systemic circulation. The BBB is composed of tight junctions, pericytes, and astrocytic end-feet, and exhibits low permeability to many lipophilic TKIs. CNS metastases are common in LCINS; however, drug concentrations in the brain often remain low because of limited penetration, poor solubility, and rapid elimination [6]. In addition, internalized drug molecules may become sequestered within endosomal or lysosomal compartments, further reducing their bioavailability in the cytoplasm.

**Figure 1:**
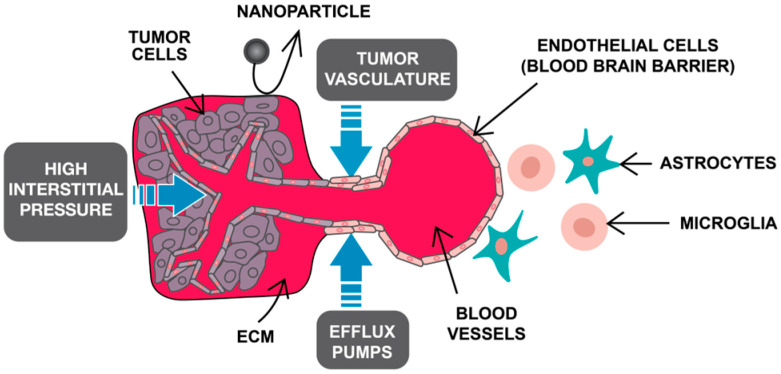
Barriers to nanocarrier delivery in LCINS. Legend: ECM—extracellular matrix; LCINS—lung cancer in never-smokers.

Figure 1 illustrates the major biological and microenvironmental barriers encountered by nanoparticles in LCINS.

### 2.3. Evolutionary Routes to Resistance

Resistance emerges through several mechanisms. Secondary kinase mutations reduce TKI binding affinity; common examples include EGFR T790M and C797S, ALK L1196M and G1202R, ROS1 G2032R, RET G810R, and MET D1228H [11,12,13,14,15,16,17]. Activation or amplification of bypass pathways, such as MET, HER2/HER3, Kirsten rat sarcoma viral oncogene homolog (KRAS), or phosphoinositide 3-kinase (PI3K), can restore downstream signaling despite continued inhibition of the primary driver [18]. Phenotypic transformation, also referred to as lineage plasticity, may lead to small-cell or squamous histology. In addition, microenvironmental factors—including fibroblast-derived hepatocyte growth factor and hypoxia—can contribute to drug resistance. The risk of resistance is increased under conditions of variable or subtherapeutic drug concentrations; therefore, sustained and homogenous drug delivery to all primary and metastatic sites is required to achieve durable clinical responses [19,20].

### 2.4. Microenvironment of Brain Metastases

Brain metastases represent a major clinical challenge in LCINS, occurring in up to 50% of patients during the course of disease [21]. The brain microenvironment differs markedly from that of primary lung tumors: it is less vascularized, characterized by a unique extracellular matrix rich in hyaluronic acid, and populated by resident immune cells such as microglia and astrocytes. The BBB not only restricts drug entry but also shapes metastatic colonization by limiting the infiltration of systemic immune cells. Tumor cells that metastasize to the brain often upregulate adhesion molecules and secrete enzymes that disrupt the basement membrane. Owing to these differences, delivery strategies must account for both systemic distribution and the specialized brain microenvironment [22].

Transferrin receptor (TfR)-mediated transcytosis and low-density lipoprotein receptor-related protein 1-mediated transport are among the few pathways available for nanoparticle entry into the brain [6]. Microglia can recognize and internalize nanoparticles; therefore, biomimetic surface coatings and anti-phagocytic “self” signals may enhance nanoparticle retention within the brain parenchyma.

Beyond physical barriers, brain metastases also establish a profoundly immunosuppressive niche. Astrocytes secrete cytokines that inhibit T-cell activity and can promote a tumor-supportive microglial phenotype. Tumor-derived exosomes contribute to the formation of a premetastatic niche by disrupting the BBB and recruiting myeloid cells into the metastatic microenvironment. In addition, hypoxia within metastatic lesions induces the upregulation of angiogenic genes and promotes epithelial–mesenchymal transition [23]. Combining nanocarriers with immunomodulators—such as Toll-like receptor agonists or stimulator of interferon genes (STING) agonists—may enhance antitumor immune responses within brain metastases [24].

**Figure 2:**
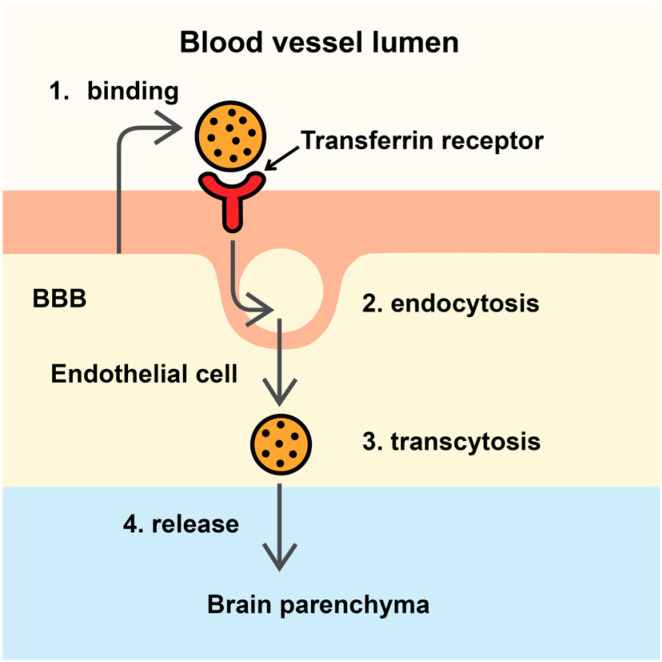
Transferrin receptor-mediated transcytosis across the BBB. **Legend**: BBB—blood–brain barrier.

Figure 2 schematically illustrates TfR-mediated transcytosis across the BBB, a key physiological pathway that can be exploited for drug delivery mechanism. Transferrin-targeted nanoparticles bind to TfR on the luminal surface of brain endothelial cells, undergo receptor-mediated endocytosis, and are transported across endothelial layer via transcytosis. Following release at the abluminal membrane, nanoparticles gain access to the brain parenchyma and deliver their therapeutic payloads.

The TfR remains one of the most important targets for therapeutic delivery across the BBB; however, significant species-specific differences pose major challenges to clinical translation.

## 3. Nanocarrier Platforms

Nanocarriers vary widely in composition, structure, and functionality, and their physicochemical properties critically influence stability, drug loading, circulation time, and tissue distribution. In the following sections, we analyze the major classes of nanocarriers relevant to LCINS. For each nanocarrier class discussed below, we explicitly delineate preclinical efficacy—using standardized endpoints such as tumor growth inhibition or survival in xenograft and orthotopic models—from evidence derived from human clinical trials, in order to facilitate translational interpretation. Selection criteria for each carrier type consider the drug payloads, ability to cross the BBB, compatibility with specific oncogenic drivers, and the required pharmacokinetic properties; lipid-based nanoparticles are prioritized for hydrophobic TKIs, polymeric or inorganic carriers for nucleic acid therapeutics, and biomimetic carriers for immunomodulatory cargos.

### 3.1. Lipid-Based Nanoparticles and Liposomes

Solid lipid nanoparticles (SLNPs) consist of a solid lipid core (e.g., glycerides or waxes) stabilized by surfactants such as phospholipids and poloxamers and are particularly well suited for the encapsulation of hydrophobic drugs [25]. An optimized formulation, termed OS4, exhibited a mean diameter of 120–180 nm, polydispersity index (PDI) below 0.3, and encapsulation efficiencies of 68–83% for messenger RNA (mRNA) [26]. Compared with standard MC3 lipid nanoparticles, OS4 increased brain delivery of nucleic acid cargo by 13.3-fold; conjugation of a Tat cell-penetrating peptide further enhanced brain delivery by 12.7-fold [26].

Although these data were obtained using mRNA-based systems, the underlying design principles are broadly applicable to small-molecule therapeutics. Nevertheless, stability during storage and lipid polymorphism remain important challenges. In addition, lipid-based carriers may activate the complement system and induce pseudoallergic reactions; these effects can be mitigated by the use of high-molecular weight polyethylene glycol (PEG) or zwitterionic surface coatings [27,28].

Liposomes are spherical vesicles composed of one or more phospholipid bilayers surrounding an aqueous core. They can encapsulate hydrophilic drugs within the aqueous core and hydrophobic drugs within the lipid bilayer. Long-circulating PEGylated liposomes loaded with isoliquiritigenin exhibited an average size of 89 nm, a PDI of 0.17, a zeta potential of −20.27 mV, and an encapsulation efficiency of 75% [29]. These formulations prolonged systemic circulation and increased brain uptake in animal models.

For brain-directed therapy, liposomes can be functionalized with targeting ligands. Internalizing cyclic arginine-glycine-aspartic acid (iRGD) peptide-modified liposomes co-loaded with osimertinib and bortezomib exhibited diameters of 125–150 nm, PDI below 0.1, and entrapment efficiencies of 42–50% for osimertinib and 25–28% for bortezomib. These dual-drug liposomes provided sustained release over approximately 48 h and significantly improved survival in intracranial xenograft models [30]. Hybrid systems that combine lipids with polymers, such as lipid-poly(lactic-co-glycolic acid) (PLGA) nanoparticles, integrate the stability of lipid carriers with the biocompatibility of polymeric systems and can achieve brain-to-plasma concentration ratios of greater than one. Despite these advantages, liposome-based formulations face challenges related to immunogenicity and stability, including complement activation, rapid clearance following opsonization, and leakage of hydrophilic payloads. A detailed discussion of these limitations and strategies to mitigate them is provided in Section 7.

### 3.2. Polymeric Micelles

Polymeric micelles consist of amphiphilic block copolymers that self-assemble into a hydrophobic core and hydrophilic shell. Direct dissolution of PEG-polylactic acid micelles for paclitaxel delivery generated particles with diameters of 178–276 nm, PDI of 0.15–0.19, and drug loading efficiencies of 30.6–52.2%, whereas microfluidic-assisted PLGA-PEG micelles produced uniform particles of 72 ± 1 nm with a PDI of 0.072 [31]. These carriers improve the solubility and half-life of hydrophobic drugs and can be functionalized with targeting ligands. However, polymeric micelles may exhibit burst release and dilution-induced disassembly in the bloodstream; cross-linking of the core or shell and the incorporation of stimuli-responsive motifs can alleviate these limitations. In addition, some micellar components, such as poly(ethylene oxide), have been associated with anti-PEG antibody formation and complement activation [32]. Considerations related to immunogenicity are discussed in Section 7.

Preclinical efficacy has been demonstrated in multiple NSCLC xenograft models. For example, pH- and redox-responsive polymeric micelles co-loaded with erlotinib or osimertinib significantly suppressed tumor growth and prolonged survival in A549 and H1975 xenograft mice compared with free drug. These effects were attributed to improved drug stability, controlled release, and enhanced tumor accumulation. In A549 models, redox- and pH-responsive erlotinib nanoparticles achieved encapsulation efficiencies exceeding 85% and produced markedly greater tumor growth inhibition than free erlotinib [33]. Collectively, these findings underscore the translational potential of stimuli-responsive micellar carriers for LCINS.

### 3.3. Chitosan–Alginate and Other Polymeric Nanoparticles

Chitosan–alginate nanoparticles are formed through ionotropic gelation between positively charged chitosan and negatively charged alginate. Alectinib-loaded chitosan–alginate nanoparticles achieved an encapsulation efficiency of approximately 97%, with a mean particle size of 161 nm and zeta potential of +21 mV. These formulations increased systemic exposure by 78% compared with free alectinib. Mucoadhesive properties and the ability to facilitate epithelial barrier crossing properties are primarily attributed to chitosan, while alginate acts as a stabilizing component of the system [34]. However, potential cytotoxicity and rapid systemic clearance remain important limitations; surface modification with PEG or zwitterionic polymers has been shown to mitigate these effects. Other polymeric nanoparticles, including PLGA, poly(β amino ester), and poly(ethylene imine), offer tunable degradation profiles and reactive functional groups for ligand conjugation. Nevertheless, challenges such as limited stability in dilute biological environments and difficulties in achieving large-scale, reproducible manufacturing persist for certain polymeric platforms [35].

### 3.4. Dendrimers and Biomimetic Carriers

Dendrimers are highly branched macromolecules characterized by a defined number of generations and internal cavities suitable for drug encapsulation. Their sizes typically range from 10 to 100 nm. Targeting ligands, imaging agents, and therapeutic moieties can be conjugated to the dendrimer surface. However, cationic dendrimers such as poly(amidoamine), may exhibit cytotoxicity due to strong interactions with cell membranes. Surface masking with red blood cell membrane or a neutral polymer can reduce toxicity and prolong systemic circulation. Exosomes and other biomimetic carriers exploit endogenous vesicles or cell membrane coatings to achieve inherent targetability, immune evasion, and, in some cases, transport across the BBB. Nevertheless, their heterogeneity, limited drug loading capacity, and challenges associated with large-scale production remain significant barriers to clinical translation [36]. Preclinical studies of dendrimer—and liposome-based gene delivery systems have demonstrated antitumor efficacy in LCINS models. SGT-53, a transferrin receptor-targeted liposomal formulation carrying a wild-type TP53 plasmid, induced dose-dependent tumor regression and improved survival in xenograft models while exhibiting acceptable safety profiles in early-phase clinical trials [37]. Similarly, BIND-014, a prostate-specific membrane antigen (PSMA)-targeted PLGA nanoparticle loaded with docetaxel, achieved higher intratumoral docetaxel concentrations, enhanced antitumor activity, and a reduced volume of distribution compared with conventional docetaxel in xenograft models of prostate, breast, and NSCLC [38,39]. Although subsequent clinical trials did not demonstrate a survival benefit, these preclinical findings underscore the potential of nanocarriers to improve the delivery and pharmacodynamic performance of chemotherapeutic and genetic payloads.

### 3.5. Mesoporous Silica Nanoparticles and Hybrid Systems

Mesoporous silica nanoparticles (MSNs) possess a large surface area and tunable mesopore sizes that enable the accommodation of small molecules and biomacromolecules. PEGylated MSNs with a diameter of approximately 25 nm and a slight positive surface charge have been shown to cross the BBB and remain in systemic circulation for more than 24 h; doxorubicin-loaded MSNs achieved a six-fold increase in brain accumulation and extended survival in glioma models. In addition, smaller MSNs (25 nm) penetrated more deeply into hypoxic tumor cores than larger particles (50 nm) [40].

However, non-degradable inorganic silica cores may accumulate in the liver and spleen. Surface modification with biodegradable polymers or the use of organosilica frameworks can mitigate this risk. Hybrid systems that combine inorganic cores with lipid or polymer shells offer controlled drug release, enhanced stability, and imaging capabilities, but they also increase formulation complexity and pose additional challenges for manufacturing and regulatory classification [41].

### 3.6. Comparative Performance and Limitations

Solid lipid nanoparticles and liposomes generally exhibit high drug-loading capacity and favorable clinical applicability; however, they may be affected by stability issues and activation of complement proteins. Polymeric micelles allow precise control over particle size and drug release profiles but can suffer from burst release and dilution-induced instability in the bloodstream. Chitosan–alginate nanoparticles offer high loading capacities and favorable biocompatibility, but often require complex surface modification to mitigate cationic cytotoxicity. Dendrimers are monodispersed, multivalent nanostructures with precise architectural control, but their cationic surface charge may be associated with cytotoxicity. PEGylated MSNs and hybrid nanoparticles provide high payload capacity and imaging functionality; however, the presence of non-degradable components may lead to long-term accumulation in non-target organs. Exosomes and other biomimetic carriers exhibit strong tissue-homing capabilities and immune evasion, but challenges related to scalable processing and efficient cargo loading remain significant.

Stimuli-responsive carriers (pH-, redox- or enzyme-sensitive micelles, polymer-drug conjugates, and exosome-like vesicles) are emerging as promising strategies to enhance on-target delivery while minimizing systemic toxicity.

Table 1 summarizes representative nanocarrier platforms for drug delivery, together with their key physicochemical properties, advantages, and limitations.

### 3.7. Emerging Platforms and Manufacturing Innovations

Although lipid- and polymer-based nanocarriers dominate current research, several emerging platforms warrant brief introduction.

Virus-like particles (VLPs) are nanostructured assemblies composed of plant- or bacteriophage-derived capsid proteins. They exhibit highly ordered geometric structures and can present multiple copies of targeting ligands on their surface [45]. VLP-based nanoparticles produced in plant systems have completed a Phase I/II clinical trial and have been approved for use in severe acute respiratory syndrome coronavirus 2 vaccines, highlighting their translational potential [46].

Although VLPs remain at a preclinical stage for LCINS drug delivery, their capacity to transport TKIs or nucleic acids, together with efficient immune evasion, positions this platform as a promising candidate for future applications. Peptide-based nanostructures represent another emerging class of carriers and consist of self-assembled amphiphilic peptides. The amino acid composition and sequence determine key properties such as cellular uptake and tissue penetration. These systems typically degrade into non-toxic metabolites but may be susceptible to proteolytic degradation, which currently limits their in vivo stability [47].

### 3.8. Patent Landscape

To provide insight into the intellectual property landscape surrounding nanocarrier-based therapies for LCINS, we surveyed patent databases of the United States Patent and Trademark Office and the European Patent Office. A growing number of patents describe lipid nanoparticles, polymeric carriers, and exosome-based platforms for the delivery of TKIs, nucleic acids, and immunomodulatory agents. However, patent expiry and limited exclusivity periods for widely used TKIs, such as erlotinib and gefitinib, may constrain the commercial viability of certain nanoformulations. This overview is intended to provide a qualitative perspective on the intellectual property landscape rather than an exhaustive analysis of individual patent claims.

Deoxyribonucleic acid (DNA) and ribonucleic acid (RNA) nanostructures exploit Watson–Crick base pairing to form cages and origami architectures capable of drug encapsulation, but their application is limited by nuclease instability and complex syntheses procedures [48,49].

Advances in manufacturing are helping to address some of these challenges. Microfluidic synthesis enables efficient and reproducible production of lipid and polymeric nanoparticles with precise size control and reduced solvent consumption, while reagent platforms have been developed for the generation of CRISPR/Cas9 ribonucleoprotein lipid nanoparticles capable of efficient in vivo genome modification [50].

In addition, three-dimensional printing and layer-by-layer assembly allow the fabrication of customizable implants and structured multilayer nanoparticles for localized and controlled therapeutic release [51].

Although these approaches remain largely experimental in the context of LCINS, they illustrate the trajectory toward next-generation precision nanomedicine and provide insights that may inform the future development of clinically relevant nanocarrier systems.

## 4. Targeted Delivery for Molecular Subtypes

To date, few clinical trials have evaluated nanocarrier-based targeted therapies specifically in LCINS. Early phase studies have primarily focused on mutation-selective EGFR inhibitors, gene therapy-based nanocarriers, and nanoparticle formulations of cytotoxic agents such as docetaxel.

BDTX-1535 is an orally bioavailable, brain-penetrant, fourth-generation EGFR/ErbB-selective inhibitor designed to overcome C797S-mediated resistance [12]. A phase I/II clinical trial (NCT05256290) has enrolled patients with EGFR-mutant NSCLC or glioblastoma to evaluate safety, dose escalation, and preliminary efficacy [52].

SGT-53 is a TfR-targeted cationic immunoliposome encapsulating plasmid DNA encoding wild-type TP53. In preclinical anti-PD-1-resistant syngeneic mouse models of NSCLC, systemic administration of SGT-53 restored p53 function, reduced immunosuppressive cell populations and mediators, and significantly enhanced the antitumor activity of anti-PD-1 therapy. Early phase clinical studies of intravenous SGT-53 in patients with refractory solid tumors demonstrated tumor-specific transgene delivery and an acceptable safety profile [37].

For nanoparticle-based docetaxel formulations, clinical efficacy in NSCLC has remained limited; however, preclinical studies consistently demonstrated improved intratumoral drug accumulation and more favorable pharmacokinetic profiles compared with conventional docetaxel.

In the context of LCINS, where durable oncogenic driver suppression and effective CNS control are critical, nanocarrier design can be tailored to the specific molecular vulnerabilities and pharmacokinetic limitations associated with each oncogenic subtype. Accordingly, this review focuses on nanocarrier strategies developed for LCINS harboring EGFR, ALK, ROS1/RET, and MET/HER2 alterations. Emphasis is placed on more recent studies, with available clinical data discussed where applicable and preclinical evidence considered when clinical results are lacking.

Table 2 summarizes the major pharmacokinetic limitations of TKIs used to target these oncogenic drivers and highlights nano-enabled strategies that have been explored to address these challenges.

### 4.1. EGFR

Activating EGFR mutations confer sensitivity to first-generation TKIs such as gefitinib and erlotinib, second-generation agents including afatinib and dacomitinib, and third-generation inhibitors such as osimertinib, almonertinib, and furmonertinib [87]. Although osimertinib exhibits improved CNS penetration compared with earlier agents, intracranial drug concentrations may remain subtherapeutic in some patients because of limited solubility and efflux transport mechanisms.

Nanocarrier-based delivery systems have been developed to address these limitations by enhancing solubility, prolonging systemic half-life, and facilitating BBB targeting. For example, transferrin-targeted nanoparticles exploit TfR overexpression in NSCLC to improve intracellular drug uptake compared with non-targeted formulations and have been shown to deliver osimertinib across the BBB, resulting in significant suppression of intracranial tumor growth in murine models [53]. In addition, iRGD-modified liposomes enhanced cellular uptake and antitumor activity in NSCLC models, including A549 xenografts, supporting their potential as delivery platforms for EGFR-targeted TKIs [53].

While polymeric micelles represent a validated strategy for improving the solubility of hydrophobic EGFR-TKIs, furmonertinib itself already demonstrates favorable pharmacokinetic properties and has shown effective brain penetration in both preclinical and clinical studies. To date, no micelle-based formulation of furmonertinib has been reported [88]. Co-delivery strategies combining EGFR-TKIs with nucleic acid therapeutics (e.g., siRNA or CRISPR constructs targeting MET or HER3) have been proposed in EGFR-mutant NSCLC to suppress bypass signaling pathways [89,90,91]. Hybrid lipid-polymer nanoparticles and related nanocarrier systems can support simultaneous drug and siRNA loading and have enabled pulmonary RNA interference delivery in lung cancer models. However, this approach has not yet been applied to osimertinib or validated as a strategy to prevent MET amplification-mediated resistance [92].

Despite current advances, achieving drug distribution within heterogeneous tumors remains challenging. LCINS lesions are frequently multifocal and exhibit marked vascular heterogeneity, with well-oxygenated regions coexisting alongside poorly perfused areas that are less accessible to nanoparticles. The concomitant use of nanocarrier-based TKIs with anti-angiogenic monoclonal antibodies, such as bevacizumab or ramucirumab, may promote vascular normalization and thereby facilitate drug penetration into tumor tissue [93]. In addition, physical modulation strategies, including mild hyperthermia combined with ultrasound have been shown to transiently increase tumor vascular permeability and may further enhance nanocarrier delivery [94,95]. EGFR-mutant lung cancers also frequently overexpress vascular endothelial growth factor, interleukin-6, and other pro-survival factors; therefore, the co-delivery of immunomodulatory agents using nanocarrier platforms could potentially augment the therapeutic efficacy of current treatment strategy [96,97].

Another emerging approach involves the integration of diagnostic and therapeutic properties within a single nanoplatform. Theranostic nanoparticles conjugated with near-infrared fluoroprobes and EGFR-TKIs may enable visualization of intratumoral drug distribution and provide early indications of therapeutic response [53]. In addition, positron-emitting radionuclide-labeled nanoparticles have been used to monitor biodistribution and systemic clearance in real time in preclinical lung cancer models and may enable assessment of brain penetration in future studies. Such imaging-guided nanoplatforms could ultimately support dose optimization and facilitate the identification of patients most likely to benefit from TKI-based nanotherapies [98].

### 4.2. ALK

ALK rearrangements, most commonly EML4–ALK fusions, are detected in approximately 3–5% of all NSCLC cases, but they are disproportionately frequent in never-smokers, younger patients, and individuals with adenocarcinoma histology [99]. Alectinib, brigatinib, and lorlatinib remain frontline ALK-TKIs. Their clinical use is limited by poor aqueous solubility and interpatient variability in CNS exposure, which is particularly relevant in LCINS given the high risk of brain metastases [5,100,101].

Alectinib-loaded chitosan–alginate nanoparticles have been developed with an entrapment efficiency of approximately 97%, a mean particle size of ~161 nm, and a favorable zeta potential. In vivo studies demonstrated improved systemic bioavailability compared with free alectinib [34].

For brigatinib, mixed polymeric micelles have been proposed as a delivery strategy, markedly enhancing drug solubility and representing a promising carrier platform for poorly soluble ALK inhibitors [102].

Because resistance frequently emerges through secondary ALK mutations, including solvent-front and compound mutations, next-generation ALK inhibitors such as repotrectinib and taletrectinib are under development. However, clinical data regarding their CNS penetration remain limited to date [103].

In parallel, co-encapsulation strategies, such as combining ALK-TKIs with epigenetic modulators or RNA/DNA-based therapeutics (e.g., p53 mRNA or CRISPR constructs), have been proposed to address bypass pathway activation or additional oncogenic alterations. Although similar nanomedicine platforms have been explored for chemotherapeutic agents and small-molecule inhibitors, they have not yet been validated in ALK-positive LCINS [104,105].

In the context of LCINS, which often presents as multifocal disease with irregular vascularization, nanocarriers offer a theoretical advantage by improving intratumoral drug distribution. In addition, lung-targeted nanocarrier systems may reduce off-target exposure and systemic toxicity, further supporting their potential translational value [105,106].

### 4.3. ROS1 and RET Fusions

ROS1 and RET fusions are relatively rare but clinically relevant oncogenic drivers in NSCLC, with a clear enrichment in never-smokers and younger patients and a distinct biological profile compared with other molecular subsets [70,71,72,73]. Selective TKIs, including entrectinib and taletrectinib for ROS1-rearranged tumors, and selpercatinib or pralsetinib for RET-altered disease, have significantly improved outcomes. However, therapeutic efficacy is frequently limited over time by the emergence of solvent-front and compound resistance mutations, such as ROS1 G2032R and RET G810R/S/C, as well as by intratumoral heterogeneity and off-target adaptive resistance mechanisms [70,71,72,73].

Nanocarrier-based formulations offer a rational strategy to further optimize these agents by enhancing aqueous solubility, prolonging systemic exposure, and enabling combination delivery. Recent work on nanotechnology-based TKI delivery systems illustrates how organic and inorganic nanoparticles, including polymeric micelles, liposomes, and hybrid carriers, can improve drug loading, pharmacokinetics, and tumor targeting, and these approaches could be adapted for ROS1- and RET-directed inhibitors [68]. Building on this concept, co-delivery strategies in which a ROS1 or RET inhibitor is combined with a mitogen-activated protein kinase (MAPK) inhibitor or other downstream pathway modulators within a single nanocarrier could, in principle, delay or suppress pathway reactivation and resistance. However, such approaches have not yet been specifically explored in ROS1- or RET-rearranged LCINS [68,70,71,72,73].

RNA-based therapeutics provide an additional layer of therapeutic modulation. Advanced hydrogel and nanogel systems have been developed to enable controlled release and protection of siRNA, microRNAs, or mRNA within the tumor microenvironment [69]. Such platforms could be engineered to co-deliver ROS1- or RET-directed TKIs together with nucleic acid therapeutics targeting bypass signaling pathways (e.g., MET or MAPK components) or restoring defective tumor suppressor function (e.g., p53 mRNA or gene-editing constructs), in line with recent nanotechnology-based delivery strategies [68,69]. At present, however, these approaches remain conceptual for ROS1- and RET-driven LCINS and have not yet been validated in dedicated preclinical models [68,69,70,71,72,73].

Because ROS1 and RET fusions are rare, most data on nanocarrier-based delivery of entrectinib, taletrectinib, selpercatinib, or pralsetinib remain preclinical or theoretical, and no nanomedicine specifically designed for ROS1- or RET-positive LCINS has yet reached clinical evaluation [68,70,71,72,73]. Multicenter collaboration and careful molecular selection, considering fusion partner, brain metastasis status, and co-mutations, will be essential to determine whether the improved pharmacokinetics, combinatorial delivery, and resistance modulation achievable with nanocarriers can translate into meaningful clinical benefit for this small but clinically important subgroup of never-smokers.

### 4.4. MET Exon 14 Skipping and HER2 Insertions

MET exon 14 skipping leads to aberrant MET signaling and increased sensitivity to MET inhibitors such as capmatinib and tepotinib, although both agents exhibit relatively short half-lives and variable CNS penetration [79]. Nanocarrier-based strategies have been explored to enhance MET inhibitor delivery, including ligand-mediated targeting approaches and pH- or redox-responsive polymeric systems [74,75,76,77,78]. In principle, such platforms could enable the co-delivery of MET TKIs together with siRNA or CRISPR-based constructs directed against bypass signaling pathways, such as KRAS or AKT (protein kinase B). This concept is supported by recent advances in RNA nanodelivery and genome-editing nanoparticle systems, although it has not yet been validated specifically in MET-driven LCINS [76,77,78].

HER2 exon 20 insertions are associated with heterogeneous responses to agents such as poziotinib, pyrotinib, and trastuzumab deruxtecan, and treatment is often limited by dose-limiting toxicity [85,86]. Nanocarrier-based strategies combining HER2 inhibitors with HER3 siRNA, PI3K inhibitors, or histone deacetylase inhibitors have been proposed to simultaneously target the primary oncogenic driver and compensatory signaling pathways, while potentially reducing off-target exposure [75,78,83]. In addition, HER3 ligand-mimetic nanobioparticles have demonstrated effective brain penetration and control of intracranial tumors, underscoring the potential of targeted delivery approaches for HER2/HER3-positive cancers [82]. More potent MET and HER2 inhibitors that retain CNS activity, including savolitinib and zongertinib, may also benefit from nanocarrier-based delivery strategies [80].

Given the frequent co-activation of the PI3K/AKT pathway in HER2-mutant LCINS, co-delivery of HER2 TKIs with PI3K inhibitors represents a rational combinatorial approach [75,78]. Antibody–drug conjugates targeting HER2 or HER3, including trastuzumab deruxtecan and patritumab deruxtecan, continue to expand therapeutic options, and integration of antibody–drug conjugate (ADC) concepts with nanocarrier platforms may further improve tumor-selective payload delivery [81,84].

### 4.5. Co-Delivery Strategies

The co-delivery of multiple therapeutic agents within a single nanocarrier is emerging as a key strategy in nano-enabled therapy for LCINS. Such systems can attenuate alternative signaling pathways and delay the onset of resistance by simultaneously targeting the primary oncogenic driver and potential escape mechanisms [64].

Core–shell nanoparticles are particularly well suited for dual loading, as the hydrophobic core can accommodate TKIs, while the hydrophilic shell can carry nucleic acids or small-molecule adjuvants. Following receptor-mediated endocytosis and cytoplasmic internalization, pH- or redox-responsive linkers can enable controlled and sequential release of both payloads [104,107].

Although co-delivery platforms specifically tailored to LCINS drivers, such as EGFR, ALK, MET, and HER2, remain largely conceptual or at a preclinical stage, recent studies have demonstrated the feasibility of combining TKIs or chemotherapeutic agents with siRNA or other nucleic acid-based therapeutics in lung cancer models, thereby supporting the broader concept of drug-gene co-delivery [76].

Advanced lipid- or polymer-based nanoparticles, including lipid-polymer hybrids and solid lipid nanoparticles, have been successfully used to deliver gene-silencing RNAs either locally or systemically, achieving significant target knockdown and antitumor effects in preclinical lung cancer models [90,108]. Nonetheless, co-delivery approaches introduce substantial formulation complexity, including challenges related to payload stability, controlled release kinetics, reproducibility, and eventual translation to the clinical setting.

**Figure 3:**
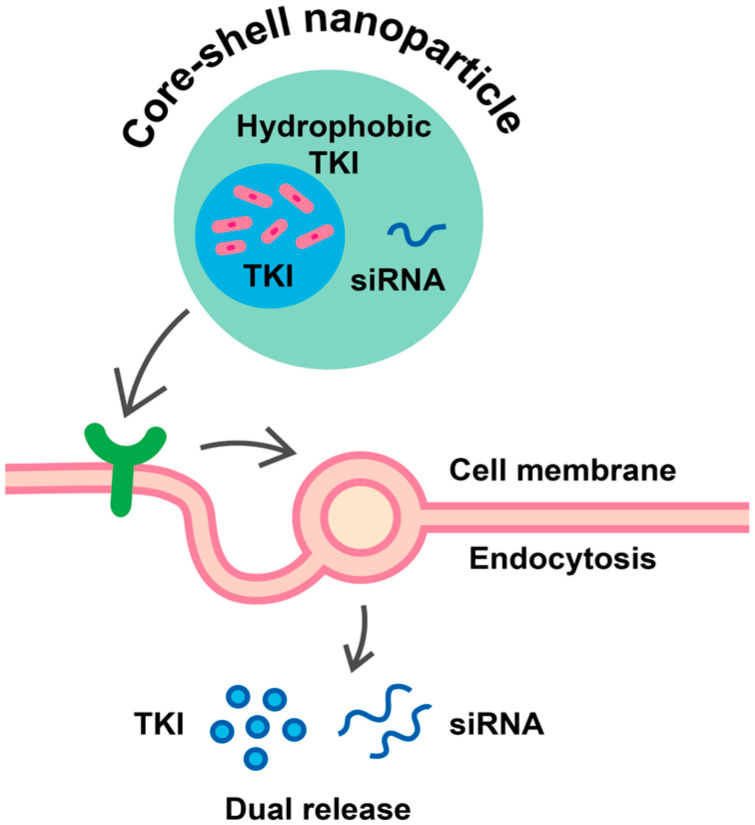
Schematic diagram illustrating a ligand-targeting core–shell nanoparticle with a hydrophobic TKI as its core and siRNA as its hydrophilic shell. Once internalized via receptor-mediated endocytosis and escaped from endosomes, there is dual release of the two therapeutic moieties into the cytoplasm. Legend: TKI—tyrosine kinase inhibitor; siRNA—small interfering ribonucleic acid.

As illustrated in Figure 3, the idealized core–shell architecture comprises a hydrophobic core hosting the TKI and a hydrophilic shell carrying the companion molecule. This design ensures solubilization and protection of both payloads while enabling temporally controlled intracellular release, an essential principle for effective combined therapy in LCINS [64,91].

Importantly, nanocarrier platforms are not intended to directly reverse genetic resistance mechanisms, such as secondary EGFR mutations, but rather to indirectly mitigate acquired resistance. By enhancing intracellular drug accumulation, limiting efflux transporter activity, improving brain penetration in CNS-progressive disease, and enabling the co-delivery of TKIs with nucleic acids or immunomodulatory agents, nanocarriers may partially overcome pharmacokinetic- and microenvironment-driven resistance in LCINS. These strategies are particularly relevant in the setting of CNS relapse and heterogeneous tumor subclones, where insufficient drug exposure is a key determinant of treatment failure.

## 5. Stimuli-Responsive Controlled Release

Nanocarriers can be designed to deliver their cargo in response to tumor microenvironment-associated stimuli, such as low pH, elevated intracellular glutathione levels, and tumor-secreted enzymes. Representative microenvironment-associated signals related to LCINS and associated cargo release properties within recent designs have been summarized in Table 3.

Controlled release is critical for maintaining therapeutic drug concentration within tumors while minimizing systemic exposure. The tumor microenvironment provides endogenous stimuli, including elevated glutathione levels and enzyme overexpression, which can be exploited to trigger on-demand drug release. Incorporation of stimulus-responsive elements into nanocarriers therefore enables spatially and temporally controlled payload release.

### 5.1. pH-Responsive Systems

The extracellular pH of solid tumors (~6.5–6.9) differs markedly from that of normal tissues (~7.4) [111]. Nanoparticles incorporating acid-labile bonds or ionizable functional groups remain stable at physiological pH but undergo structural transitions or bond cleavage under mildly acidic tumor conditions.

A recent study demonstrated that pH-sensitive nanoparticles released approximately 60% of encapsulated mitoxantrone and copper ions at pH 6.5 within 4 h, while remaining stable at physiological pH [112].

Dual pH/redox-responsive systems further enhanced efficiency, with doxorubicin-loaded carriers exhibiting up to 91% drug release at pH 6.5 in the presence of 10 mM glutathione, compared with only 29% release under physiological conditions [113].

### 5.2. Redox-Responsive Systems

Cytosolic glutathione concentrations up to 15 mM are several orders of magnitude higher than extracellular levels, which are typically below 20 µM [114,115]. Redox-responsive nanocarriers exploit this intracellular–extracellular redox gradient by incorporating disulfide or diselenide linkages that are selectively cleaved in a reducing environment. As demonstrated in dual pH/redox-responsive systems, elevated intracellular GSH levels can markedly accelerate drug release, with doxorubicin-loaded carriers showing rapid payload liberation under reducing conditions [113].

### 5.3. Enzyme-Responsive Systems

The tumor microenvironment frequently exhibits overproduction of proteolytic enzymes, including matrix metalloproteinases (MMP-2 and MMP-9) and hyaluronidase, which can selectively cleave engineered linkers within nanocarrier systems. Based on this principle, nanocarriers incorporating enzyme-cleavable peptide sequences or polysaccharide-based matrices have been developed to enable controlled drug release within the tumor microenvironment while remaining stable during systemic circulation. Enzyme-responsive delivery systems therefore represent a major class of tumor microenvironment-activated platforms and have been extensively reviewed in the recent literature [116,117,118].

### 5.4. Multi-Stage and Dual-Trigger Systems

Dual-trigger nanocarriers integrate multiple tumor-associated cues, most commonly acidic extracellular pH and elevated intracellular glutathione levels, to achieve sequential and spatially controlled drug release. A frequently employed design involves an outer pH-sensitive shell that destabilizes or is shed in response to the mildly acidic tumor microenvironment, thereby exposing a redox-sensitive core that undergoes degradation in the reducing intracellular milieu. This hierarchical response promotes cellular uptake while minimizing premature drug release during systemic circulation [113,119].

Multi-stage nanocarriers exhibiting combined pH- and redox-responsiveness have demonstrated enhanced drug release and antitumor efficacy under tumor-like conditions. For instance, nanoparticles engineered to undergo tumor-induced charge reversal and size reduction showed improved cellular internalization and controlled payload release in preclinical tumor models, supporting the concept of multi-stage trigger-mediated specificity [113].

Consistent with these findings, nanogels and polymeric carriers responsive to both acidic pH and glutathione gradients have been shown to accelerate doxorubicin release in preclinical studies, providing a mechanistic rationale for the development of dual-trigger nanocarrier systems [119].

## 6. Resistance Prevention Strategies

Secondary on-target mutations, bypass pathway activation, tumor microenvironment-mediated tolerance, and lineage plasticity represent central mechanisms of acquired resistance in oncogene-driven NSCLC, a molecular subset enriched in never-smokers [120,121,122].

Accordingly, nano-enabled resistance prevention strategies focus on (i) maintaining sustained target suppression, (ii) enabling the co-delivery of inhibitors or nucleic-acid therapeutics directed against bypass signaling pathways, and (iii) modulating epigenetic and microenvironmental factors that facilitate phenotypic switching and drug tolerance [53,123,124,125].

Sustained-release nanocarrier designs may, in principle, reduce pharmacokinetic troughs and maintain more consistent inhibitory drug exposure; however, their impact on the emergence of resistance mutations should be regarded as a hypothesis rather than an established clinical effect. Co-delivery platforms have demonstrated feasibility in NSCLC models, including nanoliposomes co-delivering osimertinib with gene-silencing payloads and multimodal nanocarrier-based approaches combining EGFR TKI with RNA therapeutics to target resistance-associated signaling pathways [123,124]. Epigenetic and lineage plasticity-associated resistance, including histologic transformation under sustained EGFR-targeted pressure, is increasingly recognized in EGFR-mutant lung cancer. This observation supports the rationale for combining TKIs with epigenetic modulators, although nano-coformulation strategies remain predominantly preclinical or conceptual strategies [126].

These resistance mechanisms provide a strong rationale for nanocarrier-based approaches that aim to improve drug exposure rather than alter the underlying oncogenic driver.

In addition, tumor microenvironment reprogramming using nanomaterials, such as macrophage- or stromal-directed modulation, has been shown to reduce regrowth following targeted therapy in lung cancer models. These findings support microenvironment-directed nanostrategies as an adjunct for delaying resistance (Table 4) [53].

## 7. Safety, Immunogenicity, and Translation

Building on the formulation and delivery strategies discussed above, this section focuses on the translational barriers that limit the clinical applicability of nanomedicine-based approaches for the treatment of LCINS.

The clinical adoption of nanomedicines depends critically on safety, manufacturability, and regulatory compliance. Immunogenicity may arise when nanocarriers activate the complement system or induce anti-polymer antibody responses [128]. PEGylation is commonly used to prolongs circulation time but can trigger anti-PEG antibodies and complement activation; however, the use of high-molecular-weight PEG or alternative zwitterionic polymers has been shown to mitigate these immune responses. Zwitterionic surface coatings reduce nonspecific protein adsorption and thrombus formation [129]. In addition, biomimetic coatings derived from erythrocyte or platelet membranes display “self” antigens that further attenuate immune recognition and clearance [130].

Biodistribution is strongly influenced by size: nanocarriers smaller than 5 nm are rapidly cleared by renal excretion, particles in the 10–200 nm range preferentially accumulate in the liver and spleen via the mononuclear phagocyte system, and larger particles may lodge in the lungs or become sequestered in the spleen [131]. Surface charge is also critical; cationic particles are cleared more rapidly whereas neutral or slightly negative particles typically exhibit prolonged circulation times [132]. Biodegradable polymers, such as PLGA, poly(β-aminoester), and chitosan, degrade into non-toxic metabolites, whereas non-degradable inorganic cores may accumulate in tissues. Regulatory agencies therefore require comprehensive characterization of particle size distribution, zeta potential, drug loading, release kinetics, and stability for each production batch [133,134,135]. Manufacturing under Good Manufacturing Practice conditions is essential to ensure uniformity and clinical safety [136].

Several nanomedicines have reached clinical use, illustrating a feasible translational pathway. Although Doxil and Abraxane are not specific to oncogene-driven NSCLC in never-smokers, they demonstrate that even relatively simple nanocarrier systems can achieve regulatory approval and large-scale manufacturing [137]. In addition, mRNA vaccines for coronavirus disease 2019, delivered via lipid nanoparticles, highlight the scalability, robustness, and safety of lipid-based carrier platforms [138]. For LCINS, successful translation will depend on achieving consistent pharmacokinetics, demonstrating efficacy in clinically relevant models, and adequately addressing off-target toxicities.

### 7.1. Manufacturing and Quality Control

The production of nanomedicines at clinical scale requires careful optimization of formulation techniques and rigorous quality control. Common preparation methods include solvent evaporation and nanoprecipitation for polymeric nanoparticles; thin-film hydration followed by extrusion for liposomes; microfluidic mixing for the generation of highly uniform micelles and lipid nanoparticles; and spray-drying or freeze-drying to improve formulations stability during storage. Each manufacturing approach influences particle size distribution, polydispersity index, and encapsulation efficiency. For example, microfluidic platforms allow precise control over flow rates and mixing times, yielding nanoparticles with narrow size distributions and high batch-to-batch reproducibility, whereas emulsion-solvent evaporation methods may result in broader size distributions but offer scalability to kilogram-scale production [139,140].

Quality control testing is a critical component of nanomedicine development. Particle size and polydispersity index can be readily determined, whereas zeta potential provides an indicator of colloidal stability. Transmission electron microscopy and atomic force microscopy are commonly used to assess particle morphology and lamellarity. Encapsulation efficiency and drug loading are quantified using high-performance liquid chromatography or ultraviolet-visible spectroscopy. Release kinetics are evaluated under physiologically relevant conditions to predict in vivo behavior. Sterility and endotoxin testing are essential to ensure safety, particularly for parenteral formulations. Stability studies examine changes in particle size, potency, and release profiles over time under different storage conditions, including refrigerated, ambient, and accelerated settings. Lyophilization with appropriate cryoprotectants can convert nanoparticle suspensions into dry powders, thereby extending shelf life and facilitating transport; however, reconstitution protocols must preserve particle integrity and performance [141].

### 7.2. Clinical Translation, Regulatory Considerations, and Case Studies

The clinical translation of nanomedicine in oncogene-driven NSCLC is uniquely influenced by the prolonged treatment duration associated with targeted therapies. In LCINS, TKIs are often administered continuously over extended periods, making cumulative immunogenicity, accelerated blood clearance phenomena, and manufacturing reproducibility clinically relevant determinants of long-term therapeutic success.

From a translational perspective, the principal challenge lies not in nanocarrier design per se, but in maintaining consistent pharmacokinetics and an acceptable safety profile across repeated dosing cycles. Variability in drug exposure or immune recognition that may be acceptable for short-course cytotoxic regimens becomes clinically limiting in LCINS, where treatment duration is typically measured in years rather than discrete cycles.

Experience from approved nanomedicines provides instructive precedents. Liposomal doxorubicin and albumin-bound paclitaxel illustrate how relatively simple and robust formulations can achieve full regulatory approval and be manufactured at large scale. Similarly, lipid nanoparticle-based mRNA vaccines have demonstrated the feasibility of industrial-scale production, global distribution, and extensive post-marketing safety evaluation. Although these products are not specifically developed for LCINS. Clinical success will therefore depend on achieving an appropriate balance between the pharmacological advantages offered by nanocarrier technologies and regulatory requirements, which are often complicated by product classification (drug, biologic, or combination product) and the need to demonstrate consistent exposure, clinical efficacy, and durable reductions in off-target toxicity.

Table 5 summarizes nanomedicine platforms that are currently advancing toward clinical evaluation for their potential application in LCINS.

Abraxane^®^, an albumin-bound paclitaxel nanoparticle, represents the only nanomedicine approved for the treatment of NSCLC, demonstrating that carrier-enabled reformulation can improve tolerability and achieve regulatory approval in lung cancer. However, its clinical use is not specific to LCINS and does not exploit the oncogene-driven biology that predominates in never-smokers [88].

BIND-014, a PSMA-targeted poly(lactic-co-glycolic acid) nanoparticle carrying docetaxel, demonstrated increased intratumoral drug accumulation but failed to improve clinical outcomes in a Phase II clinical trial in patients with NSCLC. This negative result underscores the limitations of nanoparticle targeting strategies in lung cancer when the selected target is heterogeneously expressed and not biologically linked to the dominant oncogenic drivers characteristic of LCINS [88].

SGT-53, a TfR-targeted liposomal formulation carrying a wild-type tumor protein 53 plasmid DNA, demonstrated acceptable safety and evidence of target engagement in early-phase studies that included patients with lung cancer. Although its therapeutic impact remains unproven, SGT-53 provides proof-of-concept for tumor-targeted gene delivery approaches that may be particularly relevant in LCINS, where intact delivery of biologically meaningful payloads could complement driver-directed therapies [136]. However, long-term accumulation of nanocarriers in non-target organs such as the liver, spleen, and lymph nodes, together with uncertain clearance profiles, raise safety and regulatory concerns that must be addressed prior to clinical translation.

Together, these examples illustrate that, in lung cancer—and especially in LCINS—successful clinical translation of nanomedicines depends on alignment between carrier design, biologically relevant payloads, and the molecular features of the never-smoker population, rather than on enhanced tumor accumulation alone.

## 8. Conclusions and Future Perspectives

LCINS represents a rational setting for nanotechnology-enabled precision therapy, given its dependence on dominant oncogenic drivers and the need for sustained targeted treatment, often involving CNS disease control.

However, the clinical translation of nanoformulated targeted therapies for LCINS remains modest. To date, no nanocarrier-mediated EGFR-, ALK-, or ROS1-targeted inhibitor has been initiated, and only a limited number of early-phase clinical trials have been initiated, often with small enrolment. Although preclinical studies consistently demonstrate improved pharmacokinetics, enhanced CNS drug delivery, and tumor growth inhibition, these advantages have not yet translated into meaningful survival benefits in patients. Major barriers to translation include immunogenicity (particularly associated with PEGylated systems), inefficient BBB penetration, payload leakage, manufacturing scalability, batch-to-batch variability, and regulatory complexity. In addition, the intellectual property landscape is increasingly crowded; the expiration of key lipid nanoparticle patents may further challenge the commercial viability of new nanoformulations.

Among currently explored platforms, lipid-based carriers—such as liposomes and lipid nanoparticles—exhibit the strongest translational momentum, supported by ongoing clinical evaluation of encapsulated TKIs and RNA-based therapeutics in LCINS. In contrast, polymeric, inorganic, and biomimetic nanocarriers largely remain at the preclinical stage.

## Figures and Tables

**Table 1:** Comparative performance and limitations of nanocarrier platforms.

Key Limitations	Key Advantages	Drug Loading/Half-Life Improvement	Representative Size and PDI	Platform
Lipid polymorphism; potential complement activation; limited to lipophilic drugs	High drug loading; biocompatible lipids; scalable manufacturing	Encapsulation efficiency 68–83%; prolongs circulation and improves brain delivery [26]	120–180 nm, PDI < 0.3 [26]	Solid lipid nanoparticles
Complement activation and anti-PEG antibodies [42]; potential leakage of hydrophilic payloads	Clinically validated; accommodates hydrophilic and hydrophobic drugs; ligand decoration possible	Encapsulation efficiency ~75%; increases brain uptake [29]	80–150 nm [43], PDI 0.1–0.2 [44]	PEGylated liposomes
Burst release and dilution-induced disassembly; possible immunogenicity [42]	Tunable size and release kinetics; easy functionalization	Drug loading 16–52%; extends half-life and improves solubility [31]	25–276 nm, PDI < 0.2 [31]	Polymeric micelles
Cationic toxicity; requires surface modification to reduce clearance	High drug loading; mucoadhesive; facilitates epithelial transport [34]	Encapsulation efficiency ~ 97%; AUC increase ~ 78% [34]	161 nm, positive zeta potential of +21 mV [34]	Chitosan–alginate nanoparticles
Intrinsic cytotoxicity; complex synthesis; requires neutralizing coatings [36]	Monodisperse structure; multifunctional surface groups; precise control of size [36]	High loading of small molecules and nucleic acids; extended half-life with membrane coating [36]	10–100 nm [36]	Dendrimers
Non-degradable core may accumulate; potential hepatosplenic accumulation; regulatory challenges [40]	Large surface area and pore volume; amenable to imaging; stable [40]	High loading capacity; six-fold increase in brain accumulation [40]	~25 nm, PDI < 0.1 [40]	Mesoporous silica nano-particles
Heterogeneous population; limited loading capacity; scalability and reproducibility issues	Natural homing and immune evasion; can cross biological barriers [41]	Endogenous vesicles carry proteins/RNA; prolonged circulation; improved brain delivery	30–150 nm [41]	Exosomes/ biomimetic carriers

Legend: PDI—polydispersity index; nm—nanometers; PEG—polyethylene glycol; mV—millivolts; AUC—area under the curve; RNA—ribonucleic acid.

**Table 2:** Pharmacokinetic limitations of tyrosine kinase inhibitors and nano-enabled solutions by molecular subtype.

Outcome Metrics	Illustrative Nano-Enabled Solutions	Limitations of Free TKI	Molecular Subtype
Improved brain penetration [53]; extended drug exposure [54]; may modulate MET-driven resistance [55]	Transferrin-decorated lipid nanoparticles encapsulating osimertinib [56]; iRGD-modified liposomes enhancing co-delivery and tumor penetration [57]; lipid–polymer hybrid nanoparticles enabling co-delivery of drugs and siRNA [58]	Poor solubility, rapid hepatic clearance [59], limited CNS penetration, emergence of C797S [60]	EGFR mutations
solubility improvement [61]; CNS-relevant exposure [62]; enhanced intracellular uptake [63]; reduced systemic toxicity [64]	Chitosan–alginate nanoparticles for alectinib (161 nm, 97% encapsulation, +21 mV) [34]; polymeric micelles for brigatinib [61]; brain-penetrant lorlatinib [65]	Low solubility (alectinib [34], brigatinib [66]), variable brain penetration, efflux via P-gp [67]	ALK rearrangements
Potential for improved solubility and localized release (based on nano-TKI platforms) [68]; enabling RNA- interference strategies via nanogels [69]	Conceptual nano-enabled strategies: polymeric/lipid carriers for TKI delivery [70]; nanogels enabling local release or siRNA co-delivery [69]	limited CNS penetration [71]; solvent-front mutation resistance (G2032R/G810R) [72,73]	ROS1/RET fusions
Enhanced bioavailability; sequential release triggered by pH/redox cues [74]; Blockade of pathways via co-delivery [75]	Polymeric nanoparticles- general TKI delivery [68]; hybrid silica–lipid nanoparticles [75]; co-delivery of siRNA (KRAS) [76], CRISPR [77], PI3K inhibitors [78]	Short half-life; limited brain penetration; activation of bypass pathways [79]	MET exon 14 skipping
Reduced systemic toxicity; dual inhibition of HER2 and bypass pathways [80]; potential synergy with antibody–drug conjugates [81]	Nanocarriers enabling HER3-siRNA delivery [82]; nanoparticles co-delivering epigenetic modulators (e.g., HDAC inhibitors) [83]; nanoscale carriers delivering ADC-like cytotoxic payloads [84]	Irreversible TKIs (poziotinib [85], pyrotinib [86]) cause significant toxicity; absence of validated CNS pharmacokinetic data	HER2 insertions

Legend: TKI—tyrosine kinase inhibitors; EGFR—epidermal growth factor receptor; CNS—central nervous system; iRGD—internalizing cyclic arginine-glycine-aspartic acid peptide; siRNA—small interfering ribonucleic acid; MET—mesenchymal–epithelial transition; ALK—anaplastic lymphoma kinase; P-gp—P glycoprotein; nm—nanometers; mV—millivolts; RNA—ribonucleic acid; ROS1—ros oncogene 1 receptor tyrosine kinase; RET—rearranged during transfection; KRAS—Kirsten rat sarcoma viral oncogene homolog; CRISPR—clustered regularly interspaced short palindromic repeats; PI3K—phosphoinositide 3-kinase; HER—human epidermal growth factor receptor; HDAC—histone deacetylase; ADC—antibody–drug conjugate.

**Table 3:** Stimuli responsive triggers, typical microenvironmental values, and release profiles.

Key Design Features	Representative Release Profile	Typical Trigger Values	Trigger Type
Incorporation of ionizable groups or acid labile bonds (e.g., hydrazone, imine) that undergo protonation or cleavage under mildly acidic conditions	Mildly acidic tumor extracellular pH facilitates charge conversion and improved cellular interaction and internalization of pH-responsive nanocarriers, but is stable at a physiological pH [109]	Tumor extracellular pH ~ 6.5 vs. blood pH ~ 7.4	pH- responsive
Disulfide or diselenide bonds incorporated into polymer backbones/ cross-linkers which undergo reductive cleavage in glutathione-rich intracellular environments	High cellular glutathione concentrations promote the reduction in disulfide bonds in redox-responsive nanocarriers, providing stability for circulation and an easy release of payloads inside the cell [109]	Intracellular glutathione concentrations are typically in the millimolar range, whereas extracellular levels are micromolar	Redox- responsive
Peptide linkers specifically cleavable by MMP2 and MMP9 incorporated into polymeric nanoparticles or surface coatings to achieve tumor-selective activation	Enzyme-responsive nanocarriers utilize the increased activity of MMP to allow site-specific cleavage of peptide linkers and tumor-localized activation of drug release, but remain stable in non-tumor tissue (preclinical models of lung cancer) [110]	Upregulated activity of tumor-associated proteases, particularly matrix metalloproteinases (MMP-2 and MMP-9)	Enzyme- responsive
Layered or core/shell structures where an outer pH-responsive layer reveals an inner redox or enzyme-responsive core for spatially and temporally controlled release	Multi-stage nanocarriers combine responsiveness to tumor-associated cues, enabling activation and selective intracellular delivery of the payloads, as well as limiting release during systemic circulation (conceptual and preclinical approaches) [109,110]	Combined mildly acidic extracellular pH and intracellular reductive or protease-rich environments	Multi-stage (dual-trigger)

Legend: vs.—versus; MMP—matrix metalloproteinase.

**Table 4:** Resistance mechanisms in LCINS and nano-enabled mitigation strategies.

Example	Nano-Enabled Mitigation Strategy	Biological Basis	Resistance Mechanism
Review-based evidence that CRISPR/Cas systems are being explored to overcome targeted- therapy resistance; conceptual and not yet validated as LCINS nanomedicines [125]	Sustained drug exposure via controlled-release nanocarriers; multi-agent delivery to reduce selection pressure; gene-editing approaches remain conceptual in this context	On-target mutations in the kinase domain (e.g., EGFR C797S; ALK G1202R/L1196M) reduce inhibitor binding and restore signaling despite continued TKI exposure	Secondary kinase mutations
Inhalable nanoliposomes co-delivering osimertinib and plasmid DNA for MET knockdown in an NSCLC model; preclinical proof-of-concept [123]	Co-delivery of TKIs with gene-silencing payloads targeting bypass pathways to suppress compensatory signaling	Activation or amplification of alternative receptors (e.g., MET-driven resistance to osimertinib; activation of other RTKs) restores downstream PI3K/AKT or MAPK signaling	Bypass pathway activation
Recent reviews describing histologic transformation and lineage reprogramming in EGFR-mutant NSCLC; review-based evidence [122,126]	Combination of TKIs with epigenetic modulators (HDAC or BET inhibitors) as a mechanistic strategy; nano-coformulation remains preclinical	Epigenetic reprogramming and histologic transformation (e.g., small-cell or squamous transition) under prolonged TKI pressure reduce oncogene dependence	Lineage plasticity
Superparamagnetic iron oxide nanoparticles shown to reprogram the tumor microenvironment and reduce lung cancer regrowth after crizotinib treatment; preclinical targeted-therapy model [127]	Nanoparticles designed to modulate the tumor microenvironment and limit regrowth following targeted therapy	Hypoxia, stromal cytokines, and immune suppression within the tumor microenvironment promote drug-tolerant persisted states	Microenvironment- induced tolerance

Legend: EGFR—epidermal growth factor receptor; ALK—anaplastic lymphoma kinase; TKI—tyrosine kinase inhibitor; CRISPR—clustered regularly interspaced short palindromic repeats; LCINS—lung cancer in never-smokers; MET—mesenchymal–epithelial transition; RTK—receptor tyrosine kinase; PI3K—phosphoinositide 3-kinase; AKT—protein kinase B; MAPK—mitogen-activated protein kinase; DNA—deoxyribonucleic acid; NSCLC—non-small-cell lung cancer; HDAC—histone deacetylase; BET inhibitors—bromodomain and extra-terminal motif inhibitors.

**Table 5:** Selected nanomedicine products and clinical programs relevant to lung cancer.

Key Outcomes/ Relevance to Lung Cancer	Indication and Phase	Composition/ Target	Nano- Medicine/ Program
The feasibility of nanoparticle- mediated, solvent-free chemotherapeutic approaches to lung cancer has also been demonstrated with improved tolerability and a clear path to regulatory approval, even if not limited to LCINS with oncogenic drivers [88]	Approved for NSCLC (with carboplatin)	Albumin-bound paclitaxel nanoparticles (nab-paclitaxel)	Abraxane^®^
The proof-of-concept study for The delivery of targeted nanoparticles for lung cancer has already been shown, but clinical benefit had not been achieved, underlining the need for careful targeting, efficacy, and biomarker matching [88]	Phase II clinical program including NSCLC	PSMA-targeted docetaxel PLGA nanoparticle	BIND-014
First-in-human studies Demonstrating tumor-targeting of gene delivery and acceptable safety in lung cancer patients offer proof-of-concept of nucleic acid nanomedicine in thoracic malignancy [136]	Phase I in advanced solid tumors, including lung cancer	Transferrin receptor–targeted liposome carrying TP53 plasmid DNA	SGT-53

Legend: NSCLC—non-small-cell lung cancer; LCINS—lung cancer in never-smokers; PSMA—prostate-specific membrane antigen; PLGA—poly(lactic-co-glycolic acid); TP53—tumor protein 53; DNA—deoxy-ribonucleic acid.

## Data Availability

No new data were created or analyzed in this study.
